# Common reed accumulates starch in its stem by metabolic adaptation under Cd stress conditions

**DOI:** 10.3389/fpls.2015.00138

**Published:** 2015-03-10

**Authors:** Kyoko Higuchi, Masatake Kanai, Masahisa Tsuchiya, Haruka Ishii, Naofumi Shibuya, Naoko Fujita, Yasunori Nakamura, Nobuo Suzui, Shu Fujimaki, Eitaro Miwa

**Affiliations:** ^1^Department of Applied Biology and Chemistry, Tokyo University of AgricultureSetagaya-ku, Japan; ^2^Department of Biological Production, Akita Prefectural UniversityAkita City, Japan; ^3^Medical and Biotechnological Application Division, Quantum Beam Science Center, Japan Atomic Energy AgencyTakasaki City, Japan

**Keywords:** AGPase, α-amylase, ^11^CO_2_ tracer, Cd, common reed, starch

## Abstract

In a previous study, we reported that the common reed accumulates water-soluble Cd complexed with an α-glucan-like molecule, and that the synthesis of this molecule is induced in the stem of the common reed under Cd stress. We studied the metabolic background to ensure α-glucan accumulation under the Cd stress conditions that generally inhibit photosynthesis. We found that the common reed maintained an adequate CO_2_ assimilation rate, tended to allocate more assimilated ^11^C to the stem, and accumulated starch granules in its stem under Cd stress conditions. AGPase activity, which is the rate-limiting enzyme for starch synthesis, increased in the stem of common reed grown in the presence of Cd. Starch accumulation in the stem of common reed was not obvious under other excess metal conditions. Common reed may preferentially allocate assimilated carbon as the carbon source for the formation of Cd and α-glucan complexes in its stem followed by prevention of Cd transfer to leaves acting as the photosynthetic organ. These responses may allow the common reed to grow even under severe Cd stress conditions.

## Introduction

Common reed (*Phragmites australis* [(Cav.) Trin. ex Steudel]) is resistant to various abiotic stresses and has a large biomass (Mal and Narine, 2004). Responses of common reed to NaCl-salinity (Matoh et al., 1988; Kanai et al., 2007; Liu et al., 2012), drought (Pagter et al., 2005; Liu et al., 2012), and heavy metals (Wang and Peverly, 1996; Ye et al., 1997; Bonanno, 2011) have been investigated.

Common reed is not a heavy-metal hyper-accumulator, and the Cd content in its shoot is often less than that in other wetland plants (Stoltz and Greger, 2002; Ali et al., 2004). However, common reed has been proposed as a candidate plant for rhizofiltration, because of its large biomass and ability to accumulate Cd in its roots, consequently, decreasing the Cd concentration in water (Ali et al., 2004). Therefore, the mechanisms responsible for Cd tolerance in common reed have been investigated. In the roots, the increase of phytochelatins, lignin deposition, and unknown proteins were reported to be involved in Cd tolerance (Ederli et al., 2004; Jiang and Wang, 2007). In the shoot, the activities of antioxidant enzymes and amount of glutathione were found to be increased by Cd (Fediuc and Erdei, 2002; Pietrini et al., 2003), similar to that found in other plants (Gill and Tuteja, 2011).

On the other hand, we reported a novel Cd-associated and Cd-induced molecule in the stem of the common reed (Higuchi et al., 2013). In association with Cd, this molecule is soluble and its major component is assumed to be an α-glucan (Higuchi et al., 2013). This is in contrast to rice in which decreased distribution of Cd in the soluble fraction has been reported and is thought to be responsible for increased Cd tolerance (Xiong et al., 2009). Moreover, photosynthesis is generally inhibited by excess Cd (Gallego et al., 2012; Parmar et al., 2013), and thus, it seems to be difficult for the common reed to accumulate α-glucan when grown with Cd. Furthermore, the net photosynthetic rate is reduced by Cd (Mobin and Khan, 2007; Ying et al., 2010).

In this study, we demonstrate carbon assimilation and retranslocation; the increase of AGPase activity, which is the rate-limiting enzyme for starch synthesis; and the accumulation of starch granules in the stem of common reed grown with Cd. These responses seem to be related to the supply of substrate for the synthesis of α-glucan-like molecules that are associated with Cd.

## Materials and methods

### Plant materials

Plants were cultivated in a greenhouse maintained at 24–28°C under natural light. The composition of the standard nutrient solution was 1 mM (NH_4_)_2_SO_4_, 0.5 mM KCl, 0.25 mM K_2_HPO_4_, 0.5 mM CaCl_2_, 0.5 mM MgCl_2_, 90 μM Fe-EDTA, 46 μM H_3_BO_3_, 9.2 μM MnCl_2_, 0.32 μM CuSO_4_, 0.77 μM ZnSO_4_, and 0.08 μM (NH_4_)_6_Mo_7_O_24_. The pH of the nutrient solution was adjusted to 5.5 by the addition of HCl, and the solution was changed every 3 d. Seeds of common reed collected in Hokkaido Prefecture (Japan) were purchased from Snow Brand Seed Co., Ltd. (Sapporo, Japan). The seeds were germinated on gauze floating in tap water; seedlings that grew to a height of 1–2 cm were transferred to a half-strength nutrient solution, and those that grew to a height of 5–6 cm were transferred to a full-strength nutrient solution in 5-L plastic containers. Seedlings that grew to 20 cm in height were used in subsequent experiments. For comparison of starch accumulation among several heavy metal stresses, 50 μM CdCl_2_, MnCl_2_, CuCl_2_, NiCl_2_, or ZnCl_2_ were added to the nutrient solution for 12 d. For other experiments, plants were grown with 10 μM CdCl_2_. Plants were sampled at approximately noon and stored at −80°C for subsequent assay of enzyme activity or were dried for starch measurements.

### Starch determination

The starch content of plant tissues was determined using an assay described by Wong et al. (2003). Briefly, dried plant tissues were ground using a mortar and pestle. Five to ten milligrams of ground powder was washed twice in 1 mL methanol, and then twice in 1 mL distilled water. The precipitate was suspended in 0.5 mL distilled water and heated in a boiling water bath for 2 h. The final volume was adjusted to 1 mL with distilled water. The amount of starch was determined by the method described by Bergmeyer et al. (1974). To 100 μL of the above solution, 100 μL of 50 mM Na-acetate buffer (pH 5.0) was added, followed by 50 nkat of glucoamylase (EC 3.2.1.3., *Rhizopus niveus*, Seikagaku co., Tokyo, Japan), and 5 nkat α-amylase ultrapure (EC3.2.1.1, Nippon Gene, Tokyo, Japan). This solution was mixed and incubated for 1 h at 25°C, then for 1 h at 60°C, and was then centrifuged at 10,000 × *g* for 10 min. Next, the supernatant (200 μL) was mixed with 800 μL of a solution consisting of 60 mM HEPES-KOH (pH 7.4), 5 mM MgCl_2_, 2 mM NADP, and 25 mM ATP. The amount of glucose in the solution was determined by the increase in absorbance at 340 nm after the addition of 1 mL each of hexokinase (EC 2.7.1.1, 2.8 nkat, Roche Diagnostics Japan, Tokyo, Japan) and G6P dehydrogenase (EC 1.1.1.49, 2.3 nkat, Roche Diagnostics Japan).

### Enzymatic activity

Frozen plant tissues were ground using a mortar and pestle with liquid N_2_. The protein content of samples was estimated using a Protein Assay Kit with Standard I based on the Bradford method (Bio Rad, Hercules, CA, USA). For AGPase (EC 2.7.7.27) activity, we used the method reported by Nakamura et al. (1989). Phosphoglucomutase (EC 5.4.2.2) was purchased from Sigma-Aldrich (St. Louis, MO, USA).

### Photosynthetic rate

Intact leaves from common reed plants growing in a greenhouse were used to measure the photosynthetic rate. Leaf gas exchange was measured using an open gas-exchange system (LI-6400XT; LI-COR Inc., Lincoln, NE, USA) with an integrated fluorescence chamber head (LI-6400-40 leaf chamber fluorimeter; LI-COR Inc.). The CO_2_ assimilation rate (μmol CO_2_ m^−2^·s^−1^) was measured using the stored program of the light-curve mode of LI-6400. Parameters were set as follows: TempR (relative temperature) = 20°C, CO_2_R (relative CO_2_ concentration) = 400 μmol m^−2^·s^−1^, flow = 500 μmol·s^−1^, and PQntm (light intensity and quality) = 500 μmol photon m^−2^·s^−1^ (containing 10% blue light).

### ^11^C-assimilation

We performed real-time and non-destructive imaging of ^11^C-assimilate dynamics in intact common reed plants using a positron-emitting tracer imaging system (PETIS). The upper part of young leaves from three common reed plants was inserted into a sealed acrylic compartment (inner volume: 432 mL), and other parts of the plants, including leaf sheaths, stems, and roots were set within the field of view (120 mm width × 187 mm height) of the PETIS apparatus (a modified PPIS-4800 model; Hamamatsu Photonics, Hamamatsu, Japan). ^11^CO_2_ (half-life: 20.4 min) gas was synthesized using an energetic proton beam delivered from an AVF cyclotron located at Takasaki Ion Accelerators for Advanced Radiation Application, Japan Atomic Energy Agency, following the method described by Ishioka et al. (1999). After 100 MBq of ^11^CO_2_ gas with ambient air was fed into the compartment at a flow rate of 400 mL·min^−1^, serial images of ^11^C-assimilates in plants were acquired every 10 s for 2 h using PETIS. All experiments were performed in a growth chamber under controlled conditions at 28°C and 65% relative humidity. Light was maintained at a photon flux density of approximately 400 μmol photon m^−2^·s^−1^ on the young leaf using a light-emitting diode (LED) light source (ISL-150X150-HWW; CCS Incorporated, Kyoto, Japan). Using NIH ImageJ 1.49e software (http://rsb.info.nih.gov/ij/, 2 August 2014), regions of interest (ROIs) were manually selected on each image, and the time course of ^11^C-radioactivity in the ROIs was calculated.

## Results

### Common reed can supply sufficient carbon assimilates to its stem under Cd stress

**Figure 1:**
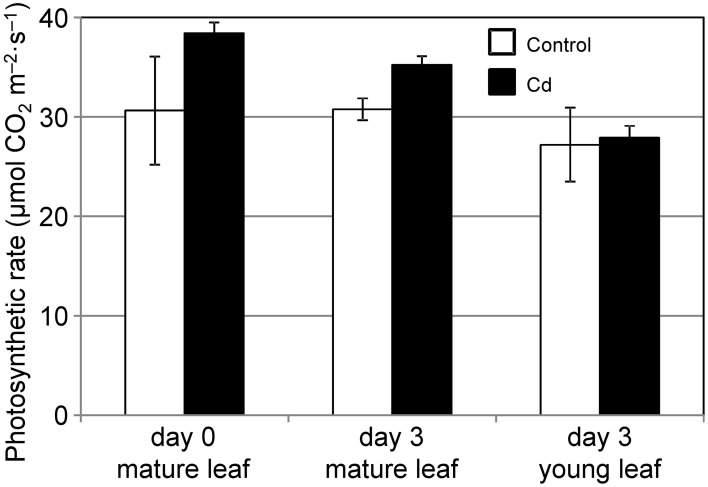
**CO_2_ assimilation rate at 500 PPFD under Cd stress**. CO_2_ assimilation rates for a mature leaf just before (day 0), and mature and young leaves 3 d after (day 3) starting 10 μ M Cd stress were measured. White bars, control plants; black bars, Cd-treated plants. We used leaves from the same three plants at day 0 and day 3. Data are represented as the mean ± SE (*n* = 3).

We analyzed the ability of common reed to assimilate and allocate carbon under conditions of 10 μ M Cd stress. By day 3 of the Cd treatment, the Cd concentration of the leaves increased noticeably (data not shown). First, we measured the CO_2_ assimilation rate under Cd stress. The CO_2_ assimilation rates of mature leaves were slightly but not significantly decreased during the 3 d of Cd stress, and were comparable to those in control leaves (Figure 1). The CO_2_ assimilation rates of young leaves were not affected by Cd (Figure 1). Although we confirmed leaf chlorosis and growth reduction in common reed induced by 2–4 weeks of Cd treatment, the leaves survived and continued to grow (data not shown).

**Figure 2:**
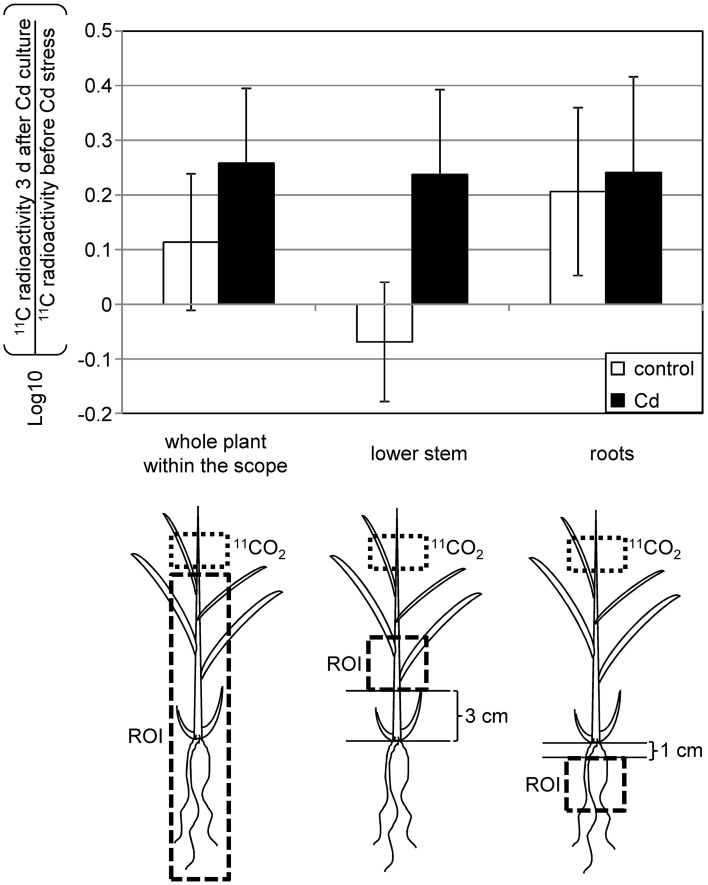
**Distribution of newly assimilated ^11^C during 3 days of culture with 10 μ M Cd**. ^11^CO_2_ was supplied to young leaves (dotted line), and radioactivity in the area under the labeled leaves was monitored (Supplementary Figure S1). The ratio of radioactivity in the regions of interest (ROI) (dashed line) before and after starting 10 μ M Cd stress was calculated for each individual plant. White bars, control plants; black bars, Cd-treated plants. Data are represented as the mean ± SE (*n* = 6).

Next, we monitored the allocation of assimilated carbon. Using a combination of ^11^C, which has a half-life of 20 min and the real-time imaging method PETIS, repeated CO_2_ assimilation and allocation of assimilates in the same plant can be detected under different conditions. The use of this method is suitable for analyzing common reed that exhibits large individual differences in shape, for example, the development of tillers or the elongation of inter nodes. We supplied ^11^CO_2_ to young leaves and monitored radioactivity for 2 h, after which each plant was grown with or without Cd for 3 d, before repeating the pulse labeling experiment (Supplementary Figure S1). Whole plant data (Figure 2) confirmed that the amount of assimilated carbon released from source leaves was not decreased by Cd stress. Carbon allocated to the stem tended to decrease in control plants during the 3-d period, whereas it tended to increase in plants grown with Cd for 3 d (Figure 2). The allocation of carbon to roots was increased in both control and Cd-treated plants during the 3-d period (Figure 2). That is, 3 d of culture with Cd seemed to increase the allocation of carbon to the stem when compared to the control plants.

### Common reed accumulates starch granules in its stem under Cd stress

Starch serves as a temporary pool of carbon. Thus far, we have observed a large number of starch granules in the stem of common reed grown with Cd. To investigate whether stress starch in stem has some special characteristics, we analyzed the shape and molecular structure of the starch present in stems. We collected starch granules from stems grown with Cd, and from control stems that contained a small amount of starch. The shapes of starch granules observed by scanning electron microscopy were similar in the control and Cd-treated plants (Supplementary Figures S2B,C). The chain-length distribution of amylopectin in these starch granules also exhibited similar patterns (Supplementary Figure S2D). Thus, Cd stress induced the accumulation of normal starch granules in the stems of common reed.

**Figure 3:**
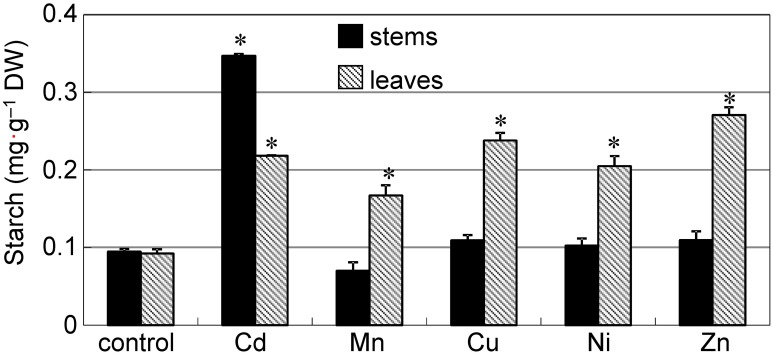
**Accumulation of starch in reed plants grown under excess heavy metal conditions**. Seedlings were divided into six groups and transplanted into normal culture solution or solutions containing an excess of heavy metals (50 μ M CdCl_2_, MnCl_2_, CuCl_2_, NiCl_2_, or ZnCl_2_), where they were then grown for 12 d. Black bar: stem. Hatched bar: leaves. Data are represented as the mean ± SE (*n* = 3). ^*^P < 0.05 indicate significant differences (according to Dunnett's test).

The accumulation of starch has previously been reported in plants under metal stress (Nyitrai et al., 2004; Zambrosi et al., 2013; Zappala et al., 2014); thus, we tested whether the accumulation of starch granules can be observed in the stem of common reed grown in the presence of other excess metals. To obtain a large number of starch granules that could be easily observed, we added 50 μ M of each heavy metal to the nutrient solution and cultured the plants for 12 d. When grown with Cd or Ni, plants were significantly shorter than the control plants (*P* < 0.05) (Supplementary Figure S3A); however, plants grew continuously and the symptoms of leaf chlorosis were mild. Thus, damage caused by Cd or Ni was not extreme when compared to plants grown with other metals. Cd, Cu, and Zn tended to accumulate more in stems than in leaves (Supplementary Figures S3B–F), especially Cd. A significant amount of starch accumulated in the leaves of all plants grown in the presence of excess heavy metals (Figure 3, hatched bars). However, the amount of starch in the stems of Cd treated plants was about three-fold higher than the amount in plants grown with other metals (Figure 3, black bars). Starch accumulation in stems grown with Cd was also clearly visible by iodine staining (Supplementary Figure S2A).

### Agpase activity is increased by Cd stress in the stem

**Figure 4:**
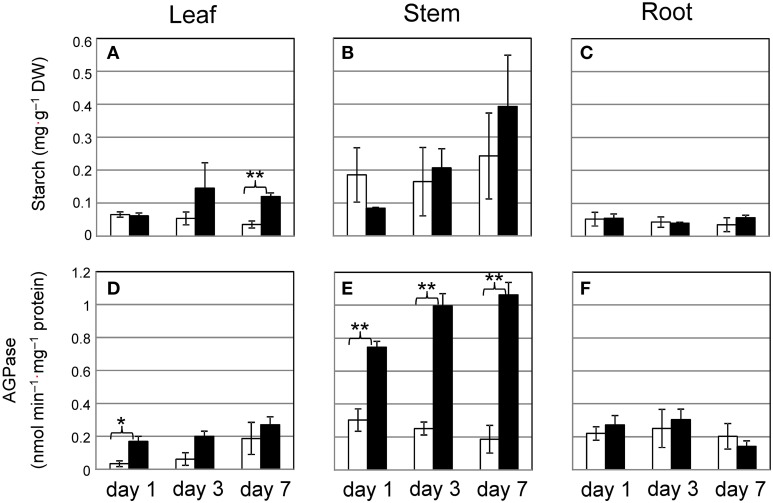
**Time course of starch accumulation and AGPase activity in common reed grown with 10 μ M Cd. (A–C)**, Starch concentration; **(D–F)**, AGPase activity. **(A,D)**, leaf; **(B,E)**, stem; **(C,F)**, root. White bars, control plants; black bars, Cd-treated plants. Data are represented as the mean ± SE (*n* = 3). ^*^P < 0.05 and ^**^P < 0.01 indicate significant differences (according to Student's *t*-test).

To investigate whether Cd triggers starch synthesis, we measured the activity of AGPase, which is the rate-limiting enzyme for starch synthesis. Although short-term culture of plants with 10 μ M Cd allows for the accumulation of only a small amount of starch granules, we tested common reed grown with 10 μ M Cd over 7 days to determine how early common reed respond to Cd. Starch levels tended to increase in stems under Cd stress, but the result was not significant (Figure 4B). The increase in AGPase activity in stems grown with Cd was significant (Figure 4E). One day of culture with Cd was sufficient to elevate AGPase activity. The amount of starch and AGPase activity in leaves tended to increase during culture with Cd, but this was not always significant (Figures 4A,D). In roots, these factors were not affected by Cd (Figures 4C,F). Common reed can grow under more severe Cd stress conditions. We confirmed the increase in starch amounts and AGPase activities in stem even under 100 μ M Cd conditions (Supplementary Figure S4).

## Discussion

In this study, we showed that common reed under Cd stress tends to allocate newly assimilated carbon to the stem when compared to that in control plants (Figure 2). In other words, the ratio of carbon distribution to the stem was not altered under Cd stress, but decreased in control plants during the 3-d experimental period (Supplementary Figure S5). The carbon source allocated to the stem might support the synthesis of α-glucan-like molecules associated with Cd, which are induced by excess Cd in the stem of common reed plants (Higuchi et al., 2013). In parallel, starch accumulates in the stem of the common reed grown with Cd. The accumulation of starch has previously been reported in plants under metal stress. Tian et al. (2010) reported that the disturbance of Na and K homeostasis caused defects in phloem loading or translocation, and consequently caused accumulation of starch in *Arabidopsis* leaves. Excess Cu disturbed the development of chloroplasts, and starch granules accumulated in cotyledons of screwbean mesquite (Zappala et al., 2014). Low-dose Pb and Ni, but not Cd, increased the starch content in detached bean leaves (Nyitrai et al., 2004), and starch granules accumulated in damaged root cells of citrus due to excess Cu (Zambrosi et al., 2013). These previous studies suggest that metal stress disturbs the export or consumption of assimilates and leads to the accumulation of starch; however, export and allocation of assimilated ^11^C from leaves to other parts of the plant was not decreased by Cd in common reed (Figure 2). Thus, starch accumulation in the stem of common reed seems to be an adaptive response, rather than a result of damage under excess Cd conditions. Abundant carbon in the stem of common reed grown with Cd could be temporarily pooled as starch and utilized to synthesize α-glucan-like molecules trapping Cd.

Both enzyme activity and supply of carbon control starch synthesis. AGPase is a key enzyme involved in starch synthesis (Preiss et al., 1991). AGPase activities increased in the stems of common reed grown with Cd within 1 day before the starch levels increased significantly (Figure 4). Common reed stimulated AGPase activity in stem even under 100 μ M Cd conditions (Supplementary Figure S4). This means that the primary carbon metabolism of common reed functions even under severe Cd stress conditions. A few studies reported the influence of mineral stress on AGPase in vegetative tissues. Chen et al. reported that AGPase activity in the leaves of rice seedlings slightly decreased under salt stress (Chen et al., 2008). Several studies discussed AGPase activity in the stems, which is important in the pooling of carbohydrates (Cook et al., 2012; Seferoglu et al., 2013), but upregulation of AGPase in the stems by mineral stress has not been reported. Whether Cd directly regulates enzymes involved in carbohydrate metabolism or indirectly influences carbon metabolism requires further study.

Zn/Cd hyper-accumulator *Picris divaricata* could maintain a CO_2_ assimilation rate at 0.2 mg·g^−1^ dry weight (DW) shoot Cd concentration (Ying et al., 2010). In the case of *Brassica juncea*, the CO_2_ assimilation rate decreased when the Cd concentration in the leaves was higher than 0.05 mg·g^−1^ DW (Mobin and Khan, 2007). In this study, less than 0.01 mg·g^−1^ DW Cd was detected in the leaves used in Figures 1, 4. The leaves of common reed may not have developed a tolerance to Cd comparable to that of hyper-accumulators, and the low Cd concentration in the leaves may be a key factor in the maintenance of photosynthesis. However, we found more than 0.05 mg·g^−1^ DW Cd in the stem used in Figure 4. Thus, amyloplasts/chloroplasts in the stem of common reed could have some mechanism for Cd tolerance.

### Conflict of interest statement

The authors declare that the research was conducted in the absence of any commercial or financial relationships that could be construed as a potential conflict of interest.

## References

[B1] Ali N. A. Bernal M. P. Ater M. (2004). Tolerance and bioaccumulation of cadmium by *Phragmites australis* grown in the presence of elevated concentrations of cadmium, copper, and zinc. Aquat. Bot. 80, 163–176 10.1016/j.aquabot.2004.08.008

[B2] Bergmeyer H. U. Bernt E. Schmidt F. Stork H. (1974). Determination with hexokinase and glucose-6-phosphate dehydrogenase, in Methods of Enzymatic Analysis, Vol. 3, eds Bergmeyer H. U. Gawehn K. (Weinheim: Verlag Chemie Weinheim), 1196–1201.

[B3] Bonanno G. (2011). Trace element accumulation and distribution in the organs of *Phragmites australis* (common reed) and biomonitoring applications. Ecotoxicol. Environ. Saf. 74, 1057–1064. 10.1016/j.ecoenv.2011.01.018 21316762

[B4] Chen H. J. Chen J. Y. Wang S. J. (2008). Molecular regulation of starch accumulation in rice seedling leaves in response to salt stress. Acta Physiol. Plant 30, 135–142 10.1007/s11738-007-0101-y

[B5] Cook F. R. Fahy B. Trafford K. (2012). A rice mutant lacking a large subunit of ADP-glucose pyrophosphorylase has drastically reduced starch content in the culm but normal plant morphology and yield. Funct. Plant Biol. 39, 1068–1078 10.1071/FP12186 32480856

[B6] Ederli L. Reale L. Ferranti F. Pasqualini S. (2004). Responses induced by high concentration of cadmium in *Phragmites australis* roots. Physiol. Plant. 121, 66–74. 10.1111/j.0031-9317.2004.00295.x 15086819

[B7] Fediuc E. Erdei L. (2002). Physiological and biochemical aspects of cadmium toxicity and protective mechanisms induced in *Phragmites australis* and *Typha latifolia*. J. Plant Physiol. 159, 265–271 10.1078/0176-1617-00639

[B8] Gallego S. M. Pena L. B. Barcia R. A. Azpilicueta C. E. Iannone M. F. Rosales E. P. (2012). Unravelling cadmium toxicity and tolerance in plants: insight into regulatory mechanisms. Environ. Exp. Bot. 83, 33–46 10.1016/j.envexpbot.2012.04.006

[B9] Gill S. S. Tuteja N. (2011). Cadmium stress tolerance in crop plants – Probing the role of sulfur. Plant Signal. Behav. 6, 215–222. 10.4161/psb.6.2.14880 21330784 PMC3121981

[B10] Higuchi K. Tsuchiya M. Nakata S. Tanabe A. Fukawa S. Kanai M.. (2013). Detoxification of cadmium (Cd) by a novel Cd-associated and Cd-induced molecule in the stem of common reed. J. Plant Physiol. 170, 1553–1560. 10.1016/j.jplph.2013.06.009 23850031

[B11] Ishioka N. S. Matsuoka H. Watanabe S. Osa A. Koizumi M. Kume T. (1999). Production of positron emitters and application of their labeled compounds to plant studies. J. Radioanal. Nucl. Chem. 239, 417–421 10.1007/BF02349523

[B12] Jiang X. Wang C. (2007). Cadmium distribution and its effects on molybdate-containinghydroxylases in *Phragmites australis*. Aquat. Bot. 86, 353–360 10.1016/j.aquabot.2007.01.002

[B13] Kanai M. Higuchi K. Hagihara T. Konishi T. Ishii T. Fujita N.. (2007). Common reed produces starch granules at the shoot base in response to salt stress. New Phytol. 176, 572–580. 10.1111/j.1469-8137.2007.02188.x 17953542

[B14] Liu Y. Li X. Liu M. Cao B. Tan H. Wang J.. (2012). Responses of three different ecotypes of reed (*Phragmites communis* Trin.) to their natural habitats: leaf surface micro-morphology, anatomy, chloroplast ultrastructure and physio-chemical characteristics. Plant Physiol. Biochem. 51, 159–167. 10.1016/j.plaphy.2011.11.002 22153253

[B15] Mal T. K. Narine L. (2004). The biology of canadian weeds. 129. *Phragmites australis* (Cav.) Trin. ex Steud. Can. J. Plant Sci. 84, 365–396 10.4141/P01-172

[B16] Matoh T. Matsushita N. Takahashi E. (1988). Salt tolerance of the reed plant *Phragmites communis*. Physiol. Plant. 72, 8–14 10.1111/j.1399-3054.1988.tb06615.x

[B17] Mobin M. Khan N. A. (2007). Photosynthetic activity, pigment composition and antioxidative response of two mustard (*Brassica juncea*) cultivars differing in photosynthetic capacity subjected to cadmium stress. J. Plant Physiol. 164, 601–610. 10.1016/j.jplph.2006.03.003 16621132

[B18] Nakamura Y. Yuki K. Park S. Y. Ohya T. (1989). Carbohydrate metabolism in the developing endosperm of rice grains. Plant Cell Physiol. 30, 833–839.

[B19] Nyitrai P. Bóka K. Gáspár L. Sárvári E. Keresztes A. (2004). Rejuvenation of ageing bean leaves under the effect of low-dose stressors. Plant Biol. 6, 708–714. 10.1055/s-2004-830385 15570476

[B20] Pagter M. Bragato C. Brix H. (2005). Tolerance and physiological responses of *Phragmites australis* to water deficit. Aquat. Bot. 81, 285–299 10.1016/j.aquabot.2005.01.002

[B21] Parmar P. Kumari N. Sharma V. (2013). Structural and functional alterations in photosynthetic apparatus of plants under cadmium stress. Bot. Stud. 54:45 10.1186/1999-3110-54-45 PMC5430381 28510881

[B22] Pietrini F. Iannelli M. A. Pasqualini S. Massacci A. (2003). Interaction of cadmiumwith glutathione and photosynthesis in developing leaves and chloro-plasts of *Phragmites australis* (Cav) Trin. ex Steudel. Plant Physiol. 133, 829–837. 10.1104/pp.103.026518 14526113 PMC219056

[B23] Preiss J. Ball K. Smith-White B. Iglesias A. Kakefuda G. Li L. (1991). Starch biosynthesis and its regulation. Biochem. Soc. Trans. 19, 539–547. 1664385 10.1042/bst0190539

[B24] Seferoglu A. B. Baris I. Morgil H. Tulum I. Ozdas S. Cevahir G.. (2013). Transcriptional reugulation of the ADP-glucose pyrophosphorylase isoforms in the leaf and the stem under long and short photoperiod in lentil. Plant Sci. 205, 29–37. 10.1016/j.plantsci.2013.01.006 23498860

[B25] Stoltz E. Greger M. (2002). Accumulation properties of As, Cd, Cu, Pb and Zn by four wetland plant species growing on submerged mine tailings. Environ. Exp. Bot. 47, 271–280 10.1016/S0098-8472(02)00002-3

[B26] Tian H. Baxter I. R. Lahner B. Reinders A. Salt D. E. Ward J. M. (2010). *Arabidopsis* NPCC6/NaKR1 is a phloem mobile metal binding protein necessary for phloem function and root meristem maintenance. Plant Cell 22, 3963–3979. 10.1105/tpc.110.080010 21193571 PMC3027173

[B27] Wang T. G. Peverly J. H. (1996). Oxidation states and fractionation of plaque iron on roots of common reeds. Soil Sci. Soc. Am. J. 60, 323–329 10.2136/sssaj1996.03615995006000010049x

[B28] Wong K. S. Kubo A. Jane J. L. Harada K. Satoh H. Nakamura Y. (2003). Structures and properties of amylopectin and phytoglycogen in the endosperm of sugary-1 mutant of rice. J. Cereal Sci. 37, 139–149 10.1006/jcrs.2002.0485

[B29] Xiong J. An L. Lu H. Zhu C. (2009). Exogenous nitric oxide enhances cadmium tolerance of rice by increasing pectin and hemicellulose contents in root cell wall. Planta 230, 755–765. 10.1007/s00425-009-0984-5 19626338

[B30] Ye Z. H. Baker A. J. M. Wong M. H. Willis A. J. (1997). Zinc, lead and cadmium tolerance, uptake and accumulation by the common reed, *Phragmites australis* (Cav.) Trin. ex Steudel. Ann. Bot. 80, 363–370 10.1006/anbo.1997.0456

[B31] Ying R. R. Qiu R. L. Tang Y. T. Hu P. J. Qiu H. Chen H. R.. (2010). Cadmium tolerance of carbon assimilation enzymes and chloroplast in Zn/Cd hyperaccumulator *Picris divaricata*. J. Plant Physiol. 167, 81–87. 10.1016/j.jplph.2009.07.005 19683362

[B32] Zambrosi F. C. B. Mesquita G. L. Tanaka F. A. O. Quaggio J. A. Mattos D. Jr. (2013). Phosphorus availability and rootstock affect copper-induced damage to the root ultra-structure of *Citrus*. Environ. Exp. Bot. 95, 25–33 10.1016/j.envexpbot.2013.07.004

[B33] Zappala M. N. Ellzey J. T. Bader J. Peralta-Videa J. R. Gardea-Torresdey J. (2014). Effects of copper sulfate on seedlings of *Prosopis pubescens* (screwbean mesquite). Int. J. Phytoremed. 16, 1031–1041. 10.1080/15226514.2013.810582 24933900 PMC4061504

