# Supplementary material for: Common reed accumulates starch in its stem by metabolic adaptation under Cd stress conditions
Source: Front Plant Sci. 2015 Mar 10;6:138. doi: 10.3389/fpls.2015.00138 (PMC4354308; doi:10.3389/fpls.2015.00138)
Supplement: Supplementary file 1 [file Presentation1.PDF]

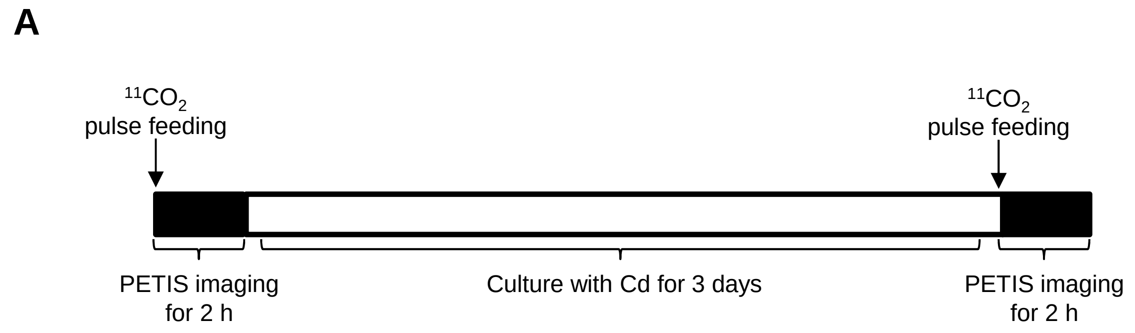

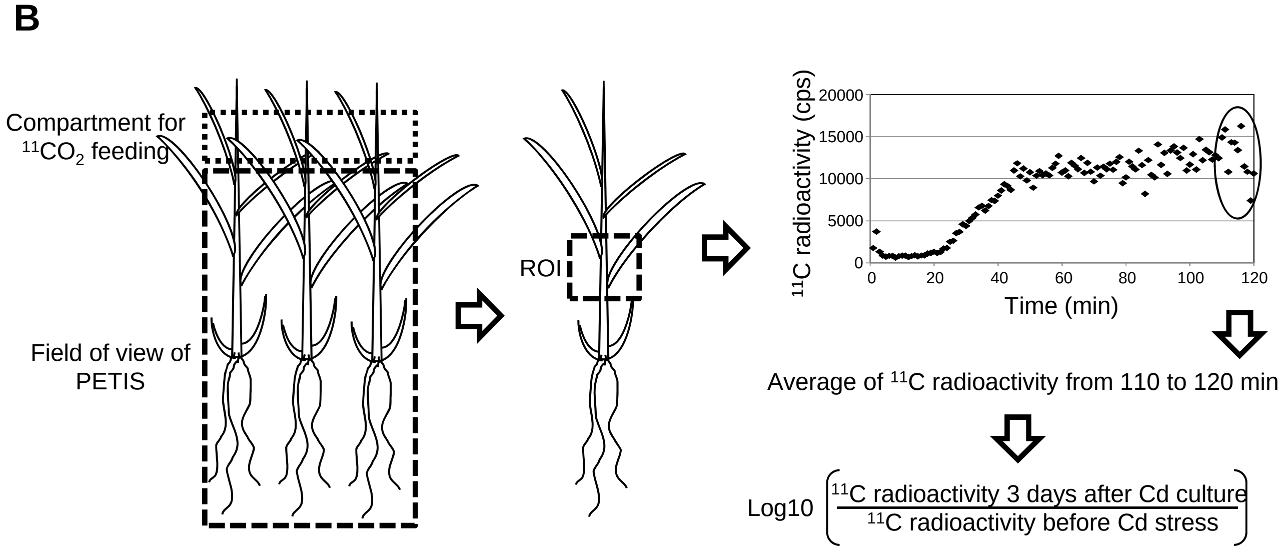

Supplementary Figure S1. Design of the ^{11}C-PETIS experiment and data analyses.

(A) Time schedule of PETIS experiment. The first pulse-feeding experiment was performed and ^{11}C radioactivity was monitored for 2 h using PETIS. Thereafter, each plant was grown with or without Cd for 3 d, and then the second pulse-feeding experiment was performed.

(B) Scheme of PETIS data analyses. First, $^{11}CO_2$ gas was fed to young leaves and serial images of ^{11}C-assimilates in plants were acquired using PETIS. Next, the time courses of ^{11}C-radioactivity in regions of interest (ROIs) were generated. Finally, the average of ^{11}C radioactivity from 110 to 120 min in each ROI was calculated and compared between the first and the second pulse-feeding experiments.

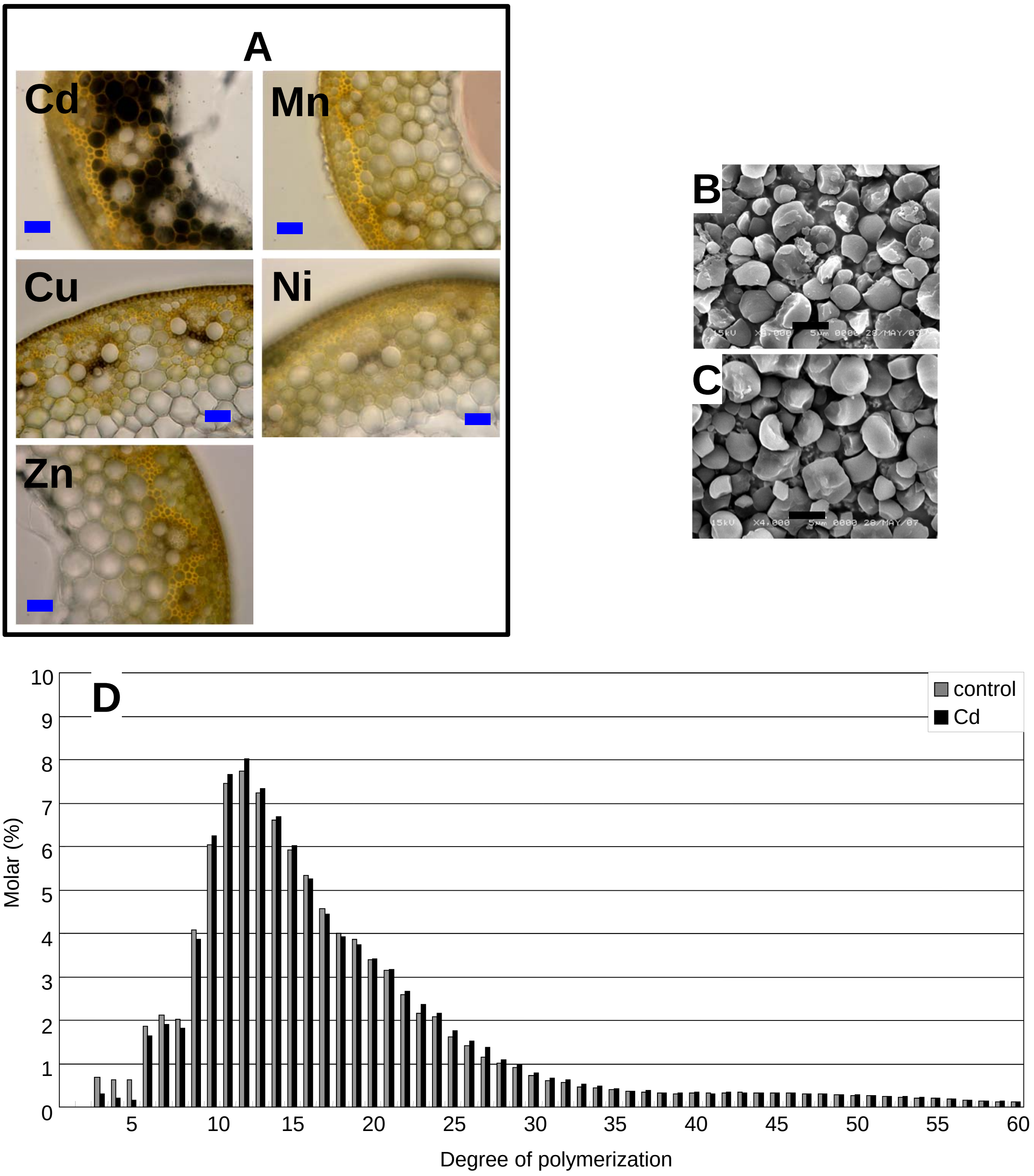

Supplementary Figure S2.
Properties of starch induced by Cd in the stem of common reed
(A) Starch produced in stems under the excess heavy metal conditions was stained with iodine solution. The growth conditions were the same as described in Figure 3. Each section was excised from the second internode of the stem. The sections were sliced transversely by using a microslicer (Dosaka EM DTK-1000, Kyoto, Japan). The thickness of the section was 80 µm. To stain the intracellular starch, the sections were incubated in an iodine solution (1% KI, 0.2% I_2) for 10 min. The incubated sections were observed with a microscope (Keyence BZ-8000, Osaka, Japan). Bars = 100 µm for all the panels. (B, C) Scanning electron microscopy of starch. B: Starch from the stem of control plants. C: Starch from the stem of Cd-treated plants. The stems were sliced transversely into 100–200-µm thick sections. The stem sections were sonicated with 10 mL of deionized water for 3 min. The solids dispersed in water were overlaid on a 41% sucrose solution and centrifuged at 15,000 $\times g$ for 1 h. The isolated granule structures were washed three times with deionized water, then observed. Bars = 5 µm for two panels.

(Continue) Supplementary Figure 2.

(D) Comparison of the chain-length distribution of amylopectin from the starches of common reed. Normalized chain length distribution of total amylopectin presented as the percentage of each peak area to the total peak areas. Gray bar: Starch from the stems of control plants. Black bar: Starch from the stems of Cd treated plants. The branched chain length distribution of the α-glucans in the granule structures was analyzed by capillary electrophoresis using the method of O'Shea et al (1998), with some modifications (Fujita et al. 2001, Plant Sci. 160, 595–602; Carbohydr. Res. 105, 1–12). The 8-amino-1,3,6-pyrene-trisulfonic acid (APTS)-labeled α-1,4-glucan chains of debranched α-glucans by isoamylase were analyzed using an eCAP N-linked oligosaccharide profiling kit and a P/ACE MDQ Carbohydrate System high-resolution capillary electrophoresis instrument equipped with a laser-induced fluorescence detector (Beckman Coulter, Inc., Fullerton, CA, USA). Data are represented as the mean.

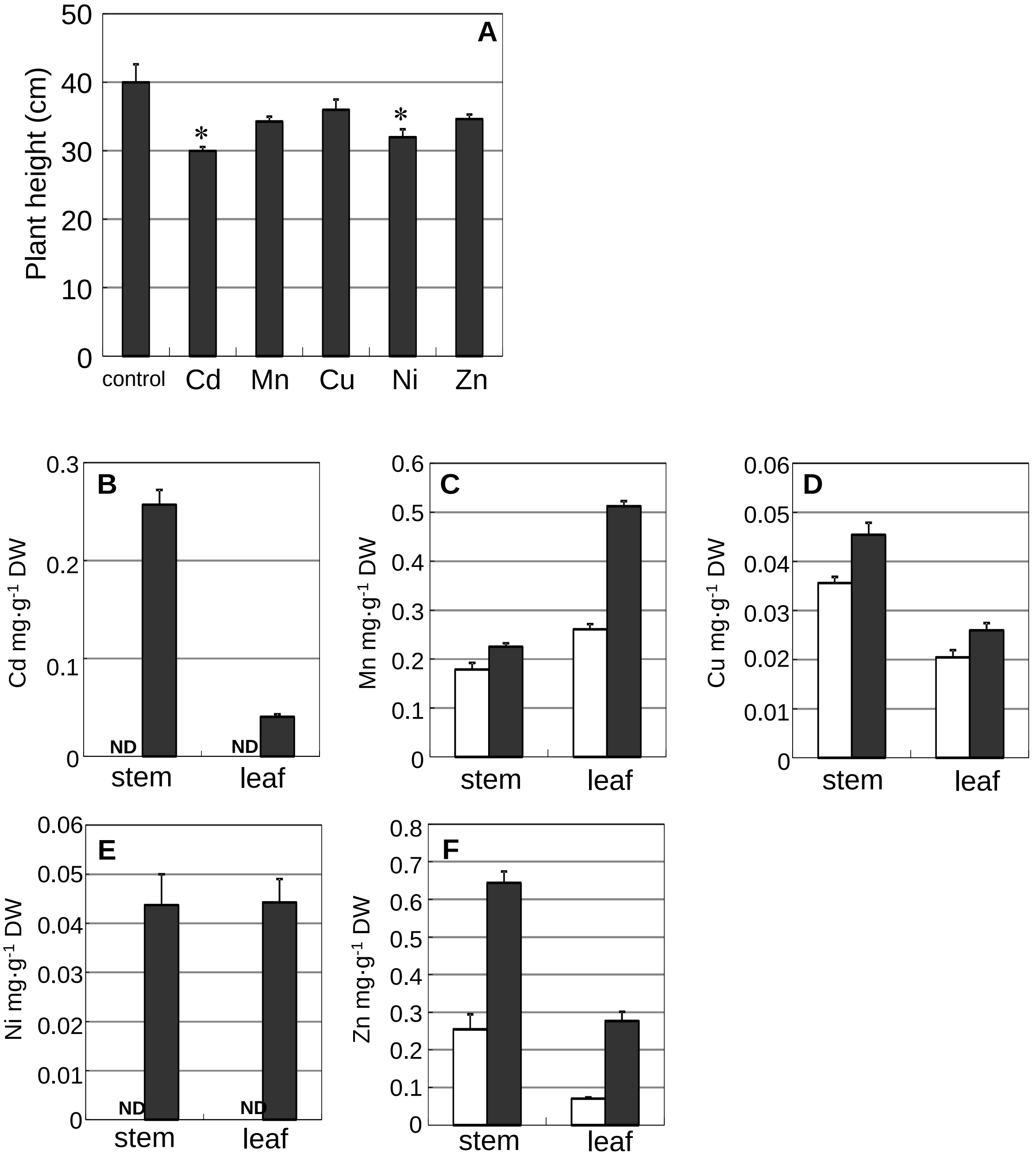

Supplementary Figure S3. Growth and accumulation of heavy metals in reed plants under excess heavy metal conditions The same plant materials shown in Figure 3 were analyzed. Data are represented as the mean ± SE (n = 3). (A) The growth of common reed under the excess heavy metal conditions. *$P < 0.05$ indicate significant differences (according to Dunnett's test). (B-F) Distribution of excess heavy metals in common reed. Black bar: heavy metal treated plant. White bar: control plant. B: Cd contents in the aerial parts of Cd-treated plants. C: Mn contents in the aerial parts of Mn-treated plants and control plants. D: Cu contents in the aerial parts of Cu-treated plants and control plants. E: Ni contents in the aerial parts of Ni-treated plants. F: Zn contents in the aerial parts of Zn-treated plants and control plants. Leaf includes leaf blade and leaf sheath. ND means "not detected."

Methods: The leaf and stem tissues were washed twice with deionized water. The plant tissues were digested with HNO_3:$HClO_4$ (4:1) at 100 to 160° C for 4 h. The total heavy metal contents of the digested products were analyzed using an atomic absorption spectrophotometer (Shimazdu AA-680, Kyoto, Japan).

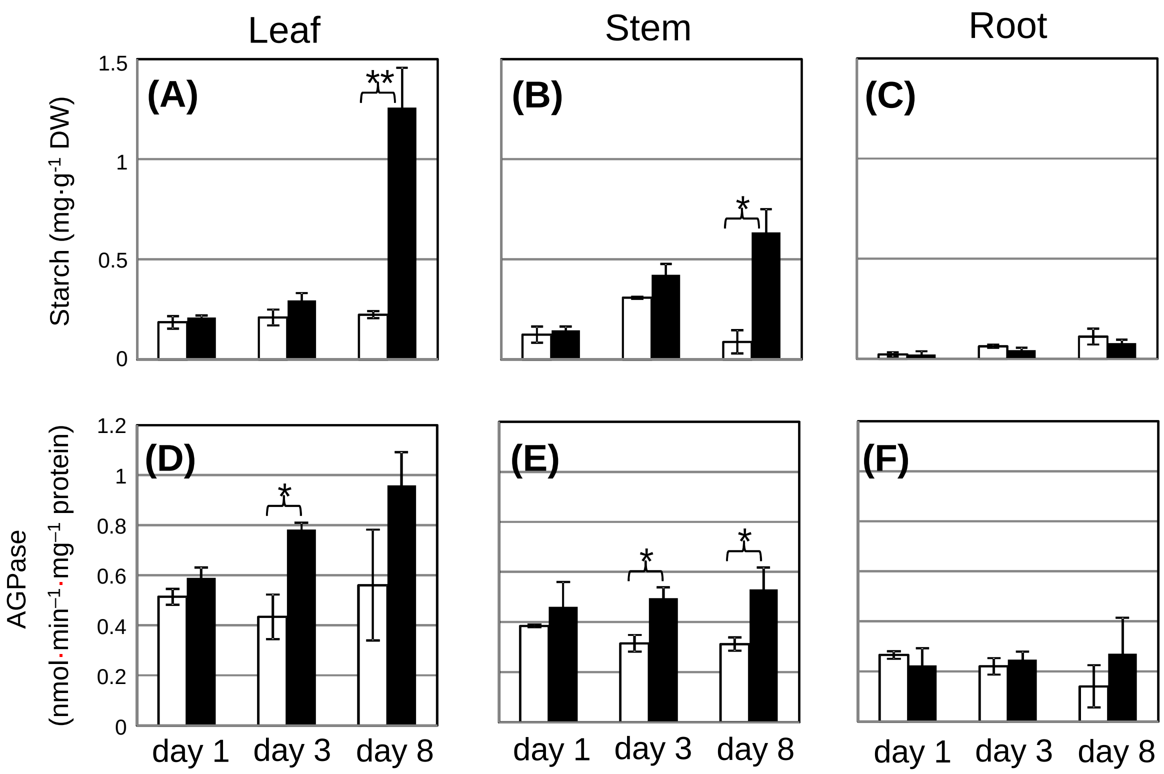

Supplementary Figure S4.
Time course of starch accumulation and AGPase activity in common reed grown with 100 μM Cd.
The experimental procedure was the same as that in Figure 4. White bars, control plants; black bars, Cd-treated plants. Data are represented as mean ± SE (n = 3). *P < 0.05 and **P < 0.01 indicate significant differences (according to Student's t-test).

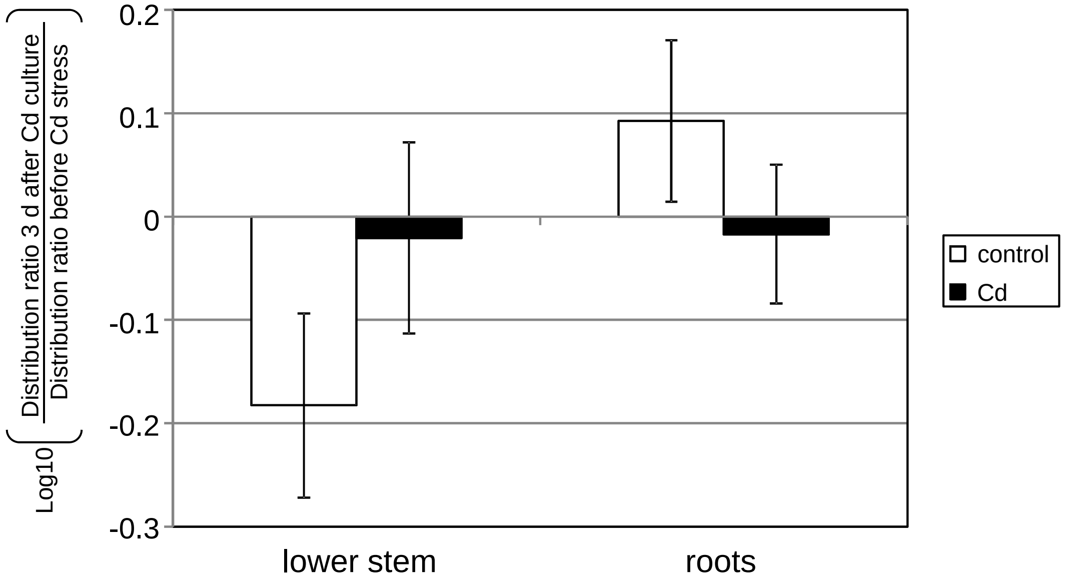

Supplementary Figure S5. The alteration of ^{11}C distribution patterns

Data presented in Figure 3 were further analyzed. First, the ratio of ^{11}C radioactivity in the regions of interest (ROIs) in the lower stem/roots to that in the whole plant ROI was calculated as the distribution ratio for each individual plant. Differences in the distribution ratio before and after 3 d of culture were then assessed. Zero means that ^{11}C distribution patterns were not altered during the 3 d. The positive value indicates an increase in ^{11}C allocation to the tissue, and the negative value indicates a decrease after 3 d in culture.
